# Integration of Sonoelastography Into the TIRADS Lexicon Could Influence the Classification

**DOI:** 10.3389/fendo.2019.00127

**Published:** 2019-03-11

**Authors:** Katarzyna Sylwia Dobruch-Sobczak, Agnieszka Krauze, Bartosz Migda, Krzysztof Mlosek, Rafał Zenon Słapa, Elwira Bakuła-Zalewska, Zbigniew Adamczewski, Andrzej Lewiński, Wiesław Jakubowski, Marek Dedecjus

**Affiliations:** ^1^Radiology Department II, The Maria Sklodowska-Curie Memorial Cancer Center and Institute of Oncology, Warsaw, Poland; ^2^Ultrasound Department, Institute of Fundamental Technological Research, Polish Academy of Science, Warsaw, Poland; ^3^Department of Imaging Diagnostics, Medical University of Warsaw, Warsaw, Poland; ^4^Department of Pathology, The Maria Sklodowska-Curie Memorial Cancer Center and Institute of Oncology, Warsaw, Poland; ^5^Department of Endocrinology and Metabolic Diseases, Medical University of Lodz, Łódź, Poland; ^6^Department of Endocrinology and Metabolic Diseases, Research Institute, Polish Mother's Memorial Hospital, Łódź, Poland; ^7^Department of Oncological Endocrinology and Nuclear Medicine, The Maria Sklodowska-Curie Memorial Cancer Center and Institute of Oncology, Warsaw, Poland

**Keywords:** TIRADS classification, thyroid cancer, thyroid nodules, ultrasonography, sonoelastography

## Abstract

**Aim:** Numerous TIRADS (Thyroid Image Reporting and Data System) classifications have been developed, and various ultrasound (US) parameters are employed in different countries. The aim of our study was to introduce risk classification and management in a native population based on the Guidelines of Polish National Societies Diagnostics and Treatment of Thyroid Carcinoma but with the addition of sonoelastography.

**Materials and Methods:** We examined prospectively 208 patients with 305 thyroid lesions employing B-mode ultrasound and sonoelastography (SE). Nodule composition, echogenicity, margins, shape, presence or absence of calcifications, thyroid capsule, nodule size were assessed using B-mode ultrasound. Moreover, sonoelastography results were presented using the Asteria scale.

**Results:** In univariate analysis, the following US features were significantly associated with malignancy: >50% solid /solid component, marked hypoechogenicity, ill-defined margins, micro and macrocalcification, taller-than wide shape, no/partial halo pattern, infiltration of the capsule and an Asteria score of 4. Multivariate logistic regression analysis of B-mode features revealed that ill-defined margins (OR 10.77), markedly hypoechogenicity (OR 5.12), microcalcifications (OR 4.85), thyroid capsule infiltrations (OR 3.2), macrocalcifications (OR 3.01), and hard lesion in SE (OR 6.85) were associated with a higher Odds Ratio (OR) for malignancy. Multivariate logistic regression analysis revealed that combining two features increases the OR and the best combination was irregular margins and Asteria scale 4 (OR 20.21). Adding a third feature did not increase the OR.

**Conclusions:** Sonoelastography increases the value risk of predicted malignancy, with consequent different approach to further clinical investigation and management. A solitary feature (Asteria 4) in a solid tumor can result in its categorization as TIRADS 4, but coexistence with high risk features allows it to be upgraded to TIRADS 5. The irregular margin was the strongest single feature which allowed for the assignment of a solid tumor into TIRADS 5 category. The highest accuracy was found by combining the features of age, margin, echogenicity (markedly hypoechoic), capsule infiltration, microcalcifications and sonoelastography (Asteria 3,4) of the tumors.

## Introduction

In Poland, 3,529 new cases of thyroid cancer were diagnosed in 2015. The annual incidence rate has increased from 3.8 per 100,000 in 2000 to 9.2 per 100,000 in 2015 (1, 2). Therefore, it is crucial to increase the accuracy of detection and surveillance in patients with thyroid disease.

Ultrasound examination (US) significantly improves the detection of thyroid nodules. According to reports in the literature, the percentage of thyroid nodules in adult patient's ranges from 33 to 68% and is higher when using higher frequency probes (1, 3). Most of these lesions are benign and reports indicate that malignant tumors below 10 mm in size are often characterized by non-aggressive behavior (2, 4), supporting the need for non- and minimally invasive diagnostic tools in patients with thyroid nodules. Currently, numerous recommendations for the risk stratification of thyroid nodules and their management are available in the literature (5–8). Most often, confirmation of the suspicious character of the nodule requires a cytological examination of the material using a fine needle aspiration biopsy (FNAB) which is the preferred the first line confirmatory investigation. Depending on the risk of malignancy in thyroid nodules, pathological verification may be recommended and a clinical decision in favor of surgery made (8). Currently, US, in combination with FNAB are considered to be the principle diagnostic tools for the diagnosis of thyroid nodules (9–12).

However, the use of US in the differentiation of malignant and benign lesions is characterized by low specificity (13). Moreover, lesions without suspicious ultrasound features, <10 mm in size, without additional clinical factors to suggest increased risk such as the presence of metastatic lymph nodes or distant metastases, neck radiation in the past, family history of thyroid cancer, appearance of a thyroid nodule before 20 or after 60 years of age or significant increase in nodule size, could be observed conservatively without proceeding with FNAB (8, 10, 14).

Currently, the basic US technique used to assess thyroid nodules is gray-scale imaging (B-mode). The features that are suspicious of malignancy in terms of US B-mode include solid composition, low or markedly low echogenicity (in relation to thyroid parenchyma and strap muscles); irregular or lobulated margins, vertical shape of the lesion (taller-than-wide) and the presence of microcalcifications and capsule infiltration (10, 15, 16). In recent years, new ultrasound techniques have appeared, such as elastography and ultrasound contrast agents to assess stiffness and micro vascularization of tissues. These techniques improve the diagnostic accuracy of US, but with some limitations (17–22).

An important step in the standardization of the assessment of the malignancy risk in thyroid nodules based on the US examination was the introduction of the TIRADS (Thyroid Imaging Reporting and Data System) classification based on the widely accepted BIRADS classification (Breast Imaging Reporting and Data System). It describes the risk of malignancy of focal lesions on an incremental scale from 0 to 6 (23). TIRADS was first proposed in 2009 (24, 25) and was the subject of further refinement in the following years (26–29). Different TIRADS classifications were published in the literature (25–27). The most recent two were proposed by the European Thyroid Association, EU-TIRADS (30), and by the American College of Radiology, ACR-TIRADS (31, 32). The above recommendations are based on a current review of the literature and expert opinion. However, there are some differences in these recommendations, even though they both utilized similar US features for predicting malignancy. In the ACR-TIRADS, experts assessed (on a scale 1–3 points, where 3 points are given for the highest risk of malignancy) the composition, echogenicity, shape, margins and the presence of echogenic foci. As an example, for the lesion to be categorized as TIRADS 5, at least 7 points are required in total. In the EU-TIRADS, it is sufficient to identify one of the high-risk US features (non-oval shape, irregular margins, microcalcification and marked hypoechogenicity) to categorize a nodule as TIRADS 5. In both guidelines sonoelastography (SE) may be used as a complementary tool for assessing nodules for FNAB but they do not precise how integrate them into risk stratification system.

In our country, Guidelines of Polish National Societies Diagnostics and Treatment of Thyroid Carcinoma recommend the use of clinical and ultrasound features, including SE (if available), in qualifying for FNAB (8).

Therefore, an attempt was made to introduce new TIRADS classification and assessment the risk of malignancy in thyroid nodules with the addition of sonoelastography in a group of polish patients.

## Materials and Methods

### Patients

In this prospective study, patients gave informed consent to participate in the research, and the institutional Review Board of the Maria Sklodowska-Curie Institute–Cancer Center, Warsaw, Poland (MSCI) approved the study. From May 2014 to November 2017, 208 patients (54 men, 154 women) with a total of 305 thyroid nodules were included in the study. The US examinations were conducted in the Department of Oncological Endocrinology and Nuclear Medicine MSCI and in Department of Imaging Diagnostics at the Medical University of Warsaw.

The inclusion criteria for the study were a thyroid nodule with suspicious features on US examination or a nodule after US-guided FNAB (according to the Guidelines of Polish National Societies) (8), with Bethesda IV-VI results and patients with Bethesda II with clinical symptoms. The exclusion criteria for the study were completely cystic lesions, lesions with eggshell calcifications, or lesions with non-diagnostic cytology results. The researchers during examination and assessment the B-mode films were blinded—not aware neither of the FNAB nor histological results. We did not use the TIRADS classification and elastography as a criterion which qualified the patient to the surgery.

### Histology

Out of 305 nodules, 126 were single nodules (126 patients), and multiple nodules (179) were found in the remaining 82 patients. Histological verification was performed in 153 thyroid nodules (including all CV-CVI lesions), and cytological confirmation was performed in 152 thyroid nodules. Histological and cytological findings were used as study endpoints. All patients with cytological confirmation of malignancy were verified by histological examination after surgery. For patients with benign FNAB results, US follow-up examination was performed within 6 months. FNABs were performed with the capillary fill, free hand technique using 22- to 24-gauge needles, and aspirates were immediately fixed in 75% ethanol and stained with haematoxylin and eosin (H&E). The cytological results were reported according to the Bethesda classification I-VI category (CI-CVI) (33). FNAB was repeated for nodules classified as CI, CIII, and small C IV nodule (<10mm). Additionally, 55/207 nodules confirmed as benign (CII) were operated on due to clinical diagnosis of compressive symptoms from an enlarged thyroid.

Cytological results (CV and CVI) were verified by an independent pathologist. The pathologist was blinded to the results of the US examination. Surgical specimens were immediately fixed in 10% buffered formalin. Representative sections from these specimens were processed and routinely stained with H&E for histopathologic (microscopic) examinations.

### Conventional B-Mode and US Examinations

Five radiologists, with experience in thyroid B-mode imaging ranging from 6 to 22 years and with experience in US elastography from 1 to 7 years performed the examinations of the thyroid glands and the cervical regions using a 5–12 MHz linear array transducer (iU22 US machine, Philips Medical Systems, Bothell, WA). During the examination, patients were in the supine position. Transverse and longitudinal sections of the lesions were investigated. During the sonoelastography, the US probe was gently placed on the thyroid and examiners avoided pressing on the thyroid with the probe, according to recommended device requirements, to reduce false-positive findings. This SE technique does not require compression and de-compression and is, therefore, operator independent. Radiologists who performed the US examinations prospectively analyzed gray-scale conventional B-mode US, color Doppler (CD) and SE (using Asteria four-point scale criteria) (17).

The following lesion features were assessed in the US examinations according to the Polish Ultrasound Society and Guidelines of Polish National Societies (8).

-composition: solid, almost solid, cystic portion <50%, cystic portion >50%, spongiform (in solid-cystic lesions the echogenicity, margins, spongiform character, angle of the wall and vascularity of the solid part were assessed);
-echogenicity: hyperechoic, isoechoic, hypoechoic—compared to surrounding thyroid tissue and markedly hypoechoic compared to strap muscle;
-margins: well-defined, irregular (spicular, jagged, angular, lobular);
-shape (taller-than-wide—tall/wide, wider-than-tall—wide/tall);
-presence or absence of calcifications (micro- or macrocalcifications) shadowing and comet- tail artifacts;
-color Doppler pattern: peripheral, central, mixed, none;
-thyroid capsule: infiltration, deformation, none;
-nodule size: volume of the nodule;

In order to calculate the volume of nodules, their shape was assumed to be ellipsoidal. The three axes of symmetry for each nodule were determined as the largest lengths of each lesion in three perpendicular directions. The three axes are referred to in the paper as “tall,” “wide,” and “long.” Finally, the volume was calculated using the ellipsoid formula.

$$\begin{array}{l} \left(\text{V}=\frac{4}{3}\;\pi \;\frac{\text{tall}}{2}\;\frac{\text{wide}}{2}\;\frac{\text{long}}{2}\;\right). \end{array}$$

Strain sonoelastography of the tumors and surrounding tissue was performed after B-mode examinations. The lesions were placed in the middle of the field of view (FOV). After 5–10 s of image stabilization, the 10 s films were recorded, and the radiologists evaluated those records containing transversal and longitudinal B-mode cross sections. Using the color map (where blue indicates hard tissue and red indicates soft tissue), the Asteria scale was reported as following:

-elasticity score (ES)-1: the nodule is displayed homogenously in red; indicating elasticity in the entire lesion;
-elasticity score (ES)-2: the nodule is displayed predominantly in green with a few blue areas/spots; indicating elasticity in a large part of the lesion;
-elasticity score (ES)-3: the nodule is displayed predominantly in blue with a few green areas/spots; indicating stiffness in a large part of the lesion;
-elasticity score (ES)-4: the nodule is displayed completely in blue (hard); indicating a lesion without elasticity;

Finally, after statistical analysis, the US features and the sonoelastography of the nodules were categorized according to independent predictors of malignancy and a new approach for classifying TIRADS risk factors was proposed.

**Figure 1:**
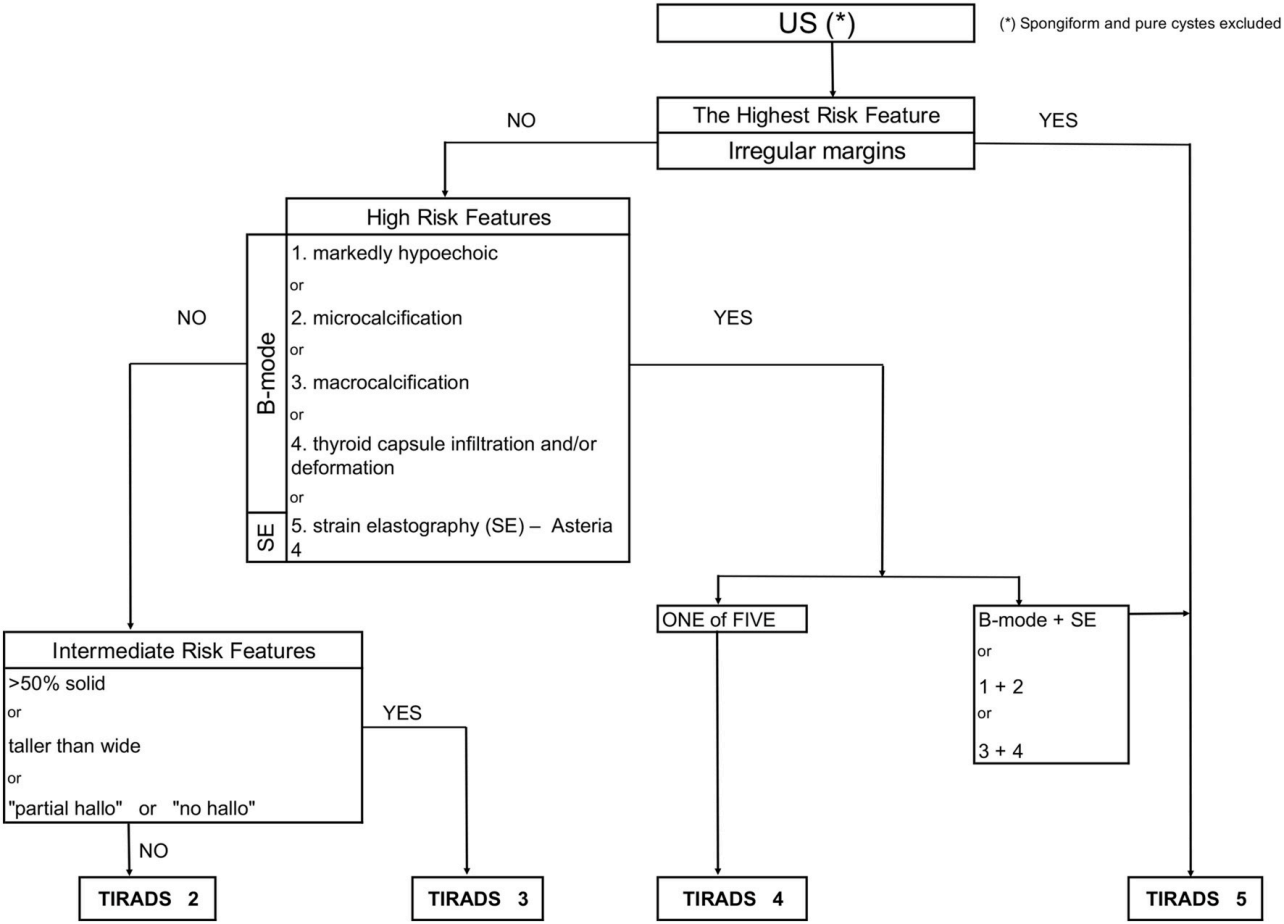
Flowchart concerning the integration of sonoelastography into the modified TIRADS classification.

We sorted the tumors into intermediate, high and highest risk of malignancy (TIRADS 3, 4 and 5 categories) according to the results from statistical analysis (accuracy) of the B-mode features and SE descriptors (Figure 1).

### Statistical Analysis

Statistical analysis was completed with the STATISTICA v 13.1 software package. Significance level α = 0.05 was used for testing of statistical hypothesis.

In the analyzed material for each continuous variable: number, arithmetic mean, standard deviation, median, lower and upper quartile, minimum value, maximum value and skewness and kurtosis coefficients were calculated. For each discrete variable, the number and the structure index were calculated. To compare means, the Student's *t*-test, Mann-Whitney *U* test and one-way analysis of variance were used. Homogeneity variance was tested using *F*-test and Brown-Forsythe's test. Scheffe's test was used as a *post-hoc* test.

Analysis of the interdependencies between a pair of discrete variables was based on the contingency table and calculations for them: row percentages, column percentages and Pearson chi-square independence test values or Fisher's exact test for four-field tables.

Odds ratios (OR) with relative 95% confidence intervals were calculated to determine the relevance of all potential predictors of outcome.

For the assessment of usability of individual US features in differentiation of benign and malignant lesions, using the contingency table, called the classification matrix, sensitivity, specificity, positive predictive value (PPV), negative predictive value (NPV) and accuracy and Youden index were calculated.

The assessment of the impact of individual independent variables on the dichotomous dependent variable based on the logistic regression model (univariate and multivariate analysis) was performed to determine independent predictors of malignancy from US features that showed statistical significance and the power of individual US features.

Finally, benign and malignant lesions were divided into two groups: lesions smaller or equal to 10 mm and those greater than 10 mm, and were compared according to US features.

## Results

### Patients

A total of 305 thyroid lesions (207 benign, 98 malignant) were identified in 208 patients with mean age 50.4 years (range 15–86 years, SD: 14.73 years). The mean age of the patients with benign lesions was 52.0 years (range 15–82 years, SD; 14.27 years). The mean age of the patients with malignant lesions was 47.8 years (range 20–86 years; SD; 15.22 years). The mean volume of all lesions was 2.67 ml (range 0.01–44.6 ml; SD; 5.51 ml); the mean volumes of malignant and benign lesions were 2.52 ml (range 0.01–28.18ml, SD; 5.08 ml) and 2.73 ml (range 0.03–44.60 ml; SD; 5.70 mm), respectively. There were 126 single nodules and 179 were in a multinodular goiter. The histological and cytological results for the 305 lesions are presented in Table 1. The Bethesda category distributions were: VI (18), V (39), IV (35), III (43), II (166), I (24).

**Table 1:** Histopathological and/or cytological results of 305 thyroid lesions.

**Pathology**	**No**.
**Benign lesions**	207
Nodular hyperplasia	181
Adenoma	18
Focal thyroiditis	8
**Malignant lesions**	98
Papillary carcinoma	81
Follicular variant	5
Follicular carcinoma	9
Medullary carcinoma	8
**Total**	305

### Conventional B-Mode US Examination

The data in Table 2 presents the characteristics of the benign and malignant nodules.

**Table 2:** The structure of the US features data; *p*-values produced by chi-square test.

**Parameter**	**Malignant lesion (%)**	**Benign lesion (%)**	***p*-value**
**Composition**			0.004
3 = >50%Solid/Solid	68 (38.20)	110 (61.80)	
2 = >50%Cystic/Almost Solid	30 (26.32)	84 (73.68)	
1 = Cystic/Spongiform	0 (0.00)	13 (100.00)	
**Echo pattern (in comparision to muscles)**			<0.001
1 = Hypoechoic	28 (65.12)	15 (34.88)	
2 = Izoechoic	16 (37.21)	27 (62.79)	
3 = Hyperechoic	54 (24.66)	165 (75.34)	
**Echo pattern (in comparision to thyroid)**			<0.001
1 = Hypoechoic	85 (39.35)	131 (60.65)	
2 = Izoechoic	9 (12.16)	65 (87.84)	
3 = Hyperechoic	4 (26.67)	11 (73.33)	
**Margins**			<0.001
0 = Ill-define	65 (67.01)	32 (32.99)	
1 = Well-define	33 (15.87)	175 (84.13)	
**Tall/wide**			0.006
1 = Tall/wide >= 1	67 (38.51)	107 (61.49)	
0 = Tall/wide < 1	31 (23.66)	100 (76.34)	
**Microcalcifications**			<0.001
1 = Present	63 (52.94)	56 (47.06)	
0 = Absent	35 (18.82)	151 (81.18)	
**Makrocalcifications**			<0.001
1 = Present	38 (51.35)	36 (48.65)	
0 = Absent	60 (25.97)	171 (74.03)	
**Color doppler pattern**			0.404
3 = Mixed	55 (33.95)	107 (66.05)	
2 = Central	11 (28.95)	27 (71.05)	
1 = Peripheral	26 (27.96)	67 (72.04)	
0 = Absent	6 (50.00)	6 (50.00)	
**Thyroid capsule**			<0.001
2 = Infiltration	12 (92.31)	1 (7.69)	
1 = Deformation	25 (43.86)	32 (56.14)	
0 = Not disturb	61 (25.96)	174 (74.04)	
**“Halo” pattern**			<0.001
2 = Partial/No	93 (36.47)	162 (63.53)	
1 = Yes	5 (10.00)	45 (90.00)	
**Asteria scale**			<0.001
4	44 (66.67)	22 (33.33)	
3	35 (35.00)	65 (65.00)	
2	16 (14.16)	97 (85.84)	
1	3 (11.54)	23 (88.46)	

It can be noticed that malignant tumors more often are solid, are taller-than-wide, are hypoechogenic, have irregular margins, have micro- and macrocalcifications, lack halo and are stiff on SE (*p* < 0.05) when compared to benign ones.

Benign lesions were significantly more cystic-solid (>50% solid component), iso-and hyperechoic, with well define margins, wider-than-tall without micro- and macrocalcifications, with halo and deformable on sonoelastography (*p* < 0.05) (Table 2).

In univariate analysis, the following US features were significantly associated with malignancy; with sensitivity, specificity and accuracy as follows: >50 solid /solid component (66.39, 46.86, 54.10%), marked hypoechogenicity (28.57, 92.75, 72.13%), ill-defined margins (66.33, 84.54, 78.69%), microcalcification (64.29, 72.95, 70.16%), macrocalcification (38.78, 82.61, 68.52%), taller-than wide shape (68.37, 48.31, 54.75%), no/partial halo pattern (94.90, 21.74, 45.25%), infiltration the capsule (37.75, 84.06, 69.18%) and Asteria 4 score (44.90, 89.37, 75.08%) (Tables 3, 4).

**Table 3:** Descriptive statistics of B-mode and elastographic parameters discriminating benign from malignant thyroid lesions (PPV, positive predictive value; NPV, negative predictive value; sensitivity, specificity, Youden index, sum-sensitivity+specificity).

**Parameter**	**Youden index**	**Sensitivity (%)**	**95% CI (sensitivity)**	**Specificity (%)**	**95% CI (specificity)**	**PPV (%)**	**95% CI (PPV)**	**NPV (%)**	**95% CI (NPV)**	**Sum**
Margins	0.51	66.33	56.51–74.91	84.54	78.99–88,83	67.01	57.16–75.56	84.13	78.56–88.47	302.01
Markedly hypoechoic	0.21	28.57	20.57–38.19	92.75	88.39–95.56	65.12	50.17–77.58	73.28	67.62–78.28	259.72
Microcalcifications	0.37	64.29	54.43–73.07	72.95	66.52–78.54	52.94	44.02–61.68	81.18	74.96–86.15	271.36
Thyroid capsule infiltration	0.22	37.76	28.79–47.64	84.06	78.46–88.42	52.86	41.32–64.10	74.04	68.08–79.23	248.71
Macrocalcifications	0.21	38.78	29.73–48.67	82.61	76.86–87.17	51.35	40.18–62.39	74.03	68.01–79.25	246.76
Asteria scale	0.34	44.90	35.43–54.75	89.37	84.43–92.88	66.67	54.66–76.84	77.41	71.69–82.25	278.34
“Halo” pattern	0.17	94.90	88.61–97.80	21.74	16.66–27.85	36.47	30.80–42.54	90.00	78.64–95.65	243.11
Composition (solid)	0.16	69.39	59.68–77.64	46.86	40.18–53.65	38.20	31.38–45.52	76.38	68.28–82.92	230.83
Tall/wide	0.17	68.37	58.62–76.73	48.31	41.59–55.09	38.51	31.60–45.91	76.34	68.37–82.80	231.52

**Table 4:** Logistic regression analysis of Asteria Scale parameter and B-mode risk factors in predicting malignancy of thyroid lesions (*p*-values were calculated by Chi-square tests).

**Parameter**	**Malignant if**	**OR**	**95% CI**	***p*-value**	**Accuracy (%)**
Margins	= 0	10.77	6.12–18.97	<0.001	78.69
Markedly hypoechoic	= 1	5.12	2.58–10.18	<0.001	72.13
Microcalcifications	= 1	4.85	2.90–8.14	<0.001	70.16
Thyroid capsule infiltration	>0	3.20	1.84–5.57	<0.001	69.18
Macrocalcifications	= 1	3.01	1.74–5.19	<0.001	68.52
Asteria Scale	= 4	6.85	3.77–12.45	<0.001	75.08
“Halo” pattern	= 2	5.17	1.97–13.53	<0.001	45.25
Composition (solid)	= 3	2.00	1.20–3.33	0.007	54.10
Tall/wide	>= 1	2.02	1.22–3.36	0.006	54,75

Using ORs (multivariate logistic regression analysis) for the association of significant predictors with the odds of malignant outcome, we determined that irregular margins (OR, 10.77; *p* < 0.01) had the highest OR. Then, marked hypoechogenicity (OR, 5.12; *p* < 0.01), microcalcifications (OR, 4.85; *p* < 0.01), thyroid capsule infiltrations (OR, 3.2; *p* < 0.01), macrocalcifications (OR, 3.01; *p* < 0.01), and hard lesion in SE (Asteria 4) (OR, 6.85; *p* < 0.01) independently predicted the malignancy (Table 4).

Using the logistic regression model, we combined the aforementioned features and demonstrated a sensitivity of 67.35%, specificity of 91.30%, PPV of 78.57%, NPV of 85.52%, accuracy of 83.61%, OR of 21.656, and a 95% CI (11.369–41.253).

### Findings of Combined Conventional B-Mode US Features and Combined With SE

To improve the accuracy of predicting malignancy in thyroid lesions, we combined the conventional US B-mode parameters with the results from SE. Multivariate logistic regression analysis revealed that combining two features: margins and Asteria (3,4 score) scale, increased OR to 20.21 (OR for margins 10.77, for Asteria scale 6.85). Also, combining another two features, echo pattern in comparison to muscles and the presence of microcalcifications significantly increased OR to 13.27 (OR for echo pattern 5.12, for microcalcifications 4.85), and macrocalcification and thyroid capsule infiltration to 7.60, respectively (OR for macrocalcification 3.00, for thyroid capsule infiltration 3.19). Adding the third feature did not increase the OR. Otherwise, combining irregular margins, which had the highest OR, with all US features did not improved statistical parameters (including OR) in differentiation the character of the lesions. The detailed findings are presented in Table 5.

**Table 5:** Logistic regression analysis of combined elastographic and B-mode parameters in predicting malignancy of thyroid lesions (OR).

**Parameter**	**OR**	**Combined OR**	**Combined OR 95% CI**	**Malignant if**	**Malignant, *n* True (+)**
Margins	10.77	20.21	10.37–39.37	0	75 (60)
Asteria Scale (4 degree)	6.85			3, 4	
Markedly hypoechoic	5.12	13.27	3.75–46.99	1	19 (16)
Microcalcifications	4.85			1	
Thyroid capsule infiltration	3.19	11.18	6.33–19.76	2, 1, 0	96 (65)
Asteria Scale (4 degree)	6.85			(3, 4), (3, 4), (4).	
Asteria Scale (4 degree)	6.85	9.45	4.71–18.95	4	51 (38)
Markedly hypoechoic	5.12			1	
Microcalcifications	4.85	8.62	4.81–15.46	1	76 (52)
Asteria Scale (4 degree)	6.85			3, 4	
Macrocalcifications	3.00	8.69	4.99–15.14	1	94 (61)
Asteria Scale (4 degree)	6.85			3, 4	
Macrocalcifications	3.00	7.63	3.26–17.86	2	
Thyroid capsule infiltration	3.19			0,1	31 (23)

We sorted the tumors into intermediate, high and highest risk of malignancy.

Logistic regression analysis showed the lowest accuracy (<55%) for three features including composition (solid or >50% solid), shape (taller-than-wide) and halo pattern (without halo or partial halo). Therefore, we proposed a TIRADS 3 (intermediate risk) category for lesions with these features. Table 6 demonstrates that in this group 4/64 tumor were malignant, and the risk of malignancy was 6.25%. In SE, all of the tumors were found to be not suspicious (Asteria 1 and 2).

**Table 6:** Risk of malignant tumors and their frequency according to B-mode features and combination of B-mode features and sonoelastography (Asteria 3, 4).

	**TIRADS–B-mode**				**Combination B-mode+Sonoelastography**		
		**Number of nodules (*n* = 255)**	**Number of malignant nodules (*n* = 94)**	**Malignancy risk (%)**	**Number of nodules**	**Number of malignant nodules**	**Malignancy risk (%)**
TIRADS 3		64	4	6.25	0	0	
	Composition >50%Solid/Solid	55	3	5.45	0	0	x
	Tall/wide	6	1	16.67	0	0	x
	“Halo” pattern partial//no	3	0	0	0	0	x
TIRADS 4		94	24	25.53	41	14	34.14
	Markedly hypoechoic	20	8	40	12	6	50
	Microcalcifications	53	12	22.64	21	5	23.81
	Capsule:infiltration; deformation	18	4	22.22	6	3	50
	Macrocalcifications	3	0	0	2	0	0
TIRADS 5		97	65	67.01	75	60	80

*Table does not include 50 tumors (including 5 malignancy) Asteria 4 scores, which were qualified into TIRADS 4 category as a single high risk feature*.

Ill-defined margins as a single feature resulted in the highest accuracy (78.69) and also highest OR (10.77)—therefore, we proposed assigning them into TIRADS 5, the category for highest risk of malignancy.

The features such as: markedly hypoechoic, microcalcifications, thyroid capsule infiltration, or macrocalcification and Asteria 4, were assigned into TIRADS 4, the high risk of malignancy category (accuracy from 68 to 76%).

The goal was to assess the influence of the SE on proposed TIRADS 3, 4, 5 category results. Initially, according to the accuracy results for B-mode and SE features, the proposed TIRADS categories were preassigned for all tumors. Then we excluded the lesions with Asteria 4 as a single feature from TIRADS 4 category, because they were associated with increased risk of malignancy (Table 6). Afterwards, we assessed the “new” risk of malignancy, adding the SE result.

Using a logistic regression model, we combined all US features with age (<34 years) to obtain the highest accuracy. Combining the features of age, margin, echogenicity (markedly hypoechoic) capsule infiltration, and microcalcifications, we demonstrated sensitivity 69.39%, specificity 92.24%, PPV 82.93%, NPV 86.55%, accuracy 85.57%, AUC 0.871, 95%CI (0.829–0.907).

Finally, malignant and benign lesions were divided into two groups: smaller or equal to 10 mm and greater than 10 mm, and were compared according to US features. In comparison of groups of the lesions measuring <10 mm in diameter only thyroid capsule infiltration or/and deformation were statistically significantly different (*p* = 0.03) (Table 7).

**Table 7:** Comparing analysis, the frequency of occurring US features of the thyroid lesion regarding maximal measurement ≤1 cm and >1 cm (*p*-values were calculated by Chi-square tests).

**Parameter**	**Analyzed feature**	**Lesion >10 mm**		**Lesion** ≤**10 mm**		***p*-value**
		**Benign (%)**	**Malignant (%)**	**Benign (%)**	**Malignant (%)**	
Composition	>50%Solid; Solid	78 (66.10)	40 (33.90)	32 (53.33)	28 (46.67)	0.098
Echo pattern	Markedly hypoechoic	11 (39.29)	17 (60.71)	4 (26.67)	11 (73.33)	0.408
Echo pattern	Hypoechoic	85 (63.43)	49 (36.57)	46 (56.10)	36 (43.90)	0.284
Margins	Ill-define	21 (35.00)	39 (65.00)	11 (29.73)	26 (70.27)	0.592
Tall/wide	>0.9565	67 (61.47)	42 (38.53)	40 (61.54)	25 (38.46)	0.993
Microcalcifications	Present	42 (48.84)	44 (51.16)	14 (42.42)	19 (57.58)	0.530
Macrocalcifications	Present	26 (46.43)	30 (53.57)	10 (55.56)	8 (44.44)	0.500
Thyroid capsule	Infiltration; Deformation	32 (52.46)	29 (47.54)	1 (11.11)	8 (88.89)	0.030
“Halo” pattern	Partial; No	111 (66.07)	57 (33.93)	51 (58.62)	36 (41.38)	0.241
Asteria Scale	3,4	64 (57.14)	48 (42.86)	23 (42.59)	31 (57.41)	0.079
Asteria Scale	4	15 (39.47)	23 (60.53)	7 (25.00)	21 (75.00)	0.239

## Discussion

The TIRADS classification was first introduced by Horvath, and the goal of this approach was to group thyroid lesions into different categories depending on the likelihood of malignancy to precisely select thyroid nodules for biopsy or to avoid unnecessary biopsy. The main objective was to standardize and simplify reporting and improve communication between radiologists and endocrinologists. The authors described 10 patterns of thyroid nodules, but this proposal was not widely accepted and applied. Therefore, other authors proposed a new classification. Park et al. introduce the equation for calculating the probability of malignancy in thyroid nodules based on 12 dichotomic US features (25).

Kwak et al. proposed a classification based on the number of suspicious US features (27). The authors proposed a TIRADS classification, which relies on counting the number of suspicious US features, beginning from TIRADS 4. The authors subdivided the category TIRADS 4 into a, b, c, where one, two, three or four suspicious features were assigned to a subcategory of category 4, respectively. They demonstrated that, using multivariate analysis, the values of fitted probabilities and risk of malignancies increased with the number of suspicious US features. Elastography was not recommended. Our results are dissimilar to the Kwak risk stratification, where the number of suspicious features higher than two did not increase the predicted risk of malignancy, but adding sonoelastography significantly increased the predicted risk of malignancy.

In 2017, two societies, the European Thyroid Association and American College of Radiology, independently published their guidelines, the EU-TIRADS and ACR TI-RADS system for risk stratification, respectively. In 2018 in Poland, recommendations according to the Guidelines of Polish National Societies prepared on the initiative of the Polish Group for Endocrine Tumors were released. They contain the clinical and US suspicious features of thyroid nodules and indications for biopsy, but the TIRADS classification is not followed in routine practice by endocrinologists and radiologists.

Our results demonstrate that inclusion of sonoelastography suspicious features (Asteria 3,4) in the TIRADS classification system increases the risk for malignancy in TIRADS 4 and TIRADS 5 category. Unfortunately, in TIRADS 3 lesions, the inclusion of elastography features did not increase the risk of the malignancy (all lesions had sonoelastography scores of Asteria 1 or 2). There were four false negative results for elastography in this group. Therefore, for a TIRADS 3 (intermediate risk) category, FNAB is still recommended upon B-mode features finding (mean risk of malignancy 6.25%) is advocated regardless of sonoelastography results.

Ill-defined margins as a single feature resulted in the highest accuracy and highest OR (10.77)—therefore, we proposed assigning them into TIRADS 5, the category with the highest risk of malignancy, and recommended performing FNAB. When combining this features with sonoelastography (Asteria 3, 4 scores) the risk of malignancy increased even more (from 67.01 to 80.00%).

As an example, adding sonoelastography results (Asteria scale 3, 4) to ill-defined margins, reduced false positive results from 32 tumors, for ill-defined margin alone, to 10 tumors (Tables 2, 5).

The occurrence of at least one of the high-risk features in a nodule should lead to consideration of assignment to TIRADS 4 category and performance of an FNAB should be considered.

The combined analyses of SE (Asteria 3, 4 scores) with the following high risk TIRADS 4 category B-mode features: infiltration of the capsule, markedly hypoechoic microcalcification and macrocalcification, resulted in an increased prediction of malignancy using the risk of malignancy (the risk of malignancy increased from 25.53 to 34.14%). Also, the combination of two B-mode features, such as marked hypoechogenicity with microcalcification (OR = 13.27) or macrocalcification and thyroid capsule infiltration (OR 7.60) increased the OR—therefore, we recommended a classification of TIRADS5 category for tumors with these features independently, based on the size of the lesions. In the case of a non-diagnostic, or negative result, FNAB should be repeated.

Our results and proposed algorithm for TIRADS suggest that sonoelastography could be integrated into the TIRADS classification system and used in daily practice, and also as an independent risk predictor in daily practice.

If nodules are predominantly cystic or spongiform and have no features of intermediate or high risk of malignancy, then TIRADS 2 category is appropriate and surveillance recommended (7, 32).

In the EU-TIRADS, if at least one of the high-risk features: non-oval shape, irregular margins, microcalcifications or marked hypoechogenicity is present, it is recommended that the lesion should be classified into the high-risk category of EU-TIRADS 5. Only a mildly hypoechoic pattern without any feature of high risk is recommended for classification as EU-TIRADS 4 category with intermediate risk, and an FNAB is recommended for nodules >15 mm. Our results are partially concordant with the EU-TIRADS if the suspicious US B-mode features are analyzed. However, we suggest dividing them into intermediate, high and the highest risk of malignancy.

Haugen BR at al. published an ATA (American Thyroid Association) risk stratification scale of thyroid nodules, dividing them into 5 categories (benign, very low suspicion, low suspicion, intermediate suspicion and high suspicion) (7). Our results are only partially compatible with the ATA recommendations. In our study, similar to the Haugen et al. study, the group of highly suspicious features include: ill-defined margin, hypoechogenicity, microcalcifications and extra-thyroidal extension. We achieved an inconsistent result with shape (taller-then-wide), >50% solid tumor composition and halo “pattern”(partial or no), which we categorized it into intermediate risk group, and despite the difference in the risk stratification the recommendation for the biopsy was similar. In ATA guidelines shape (taller-then-wide) in solid hypoechoic or solid hypoechoic component of a partially cystic nodule is the high suspicion features group. In the most recent published paper comparing the different TIRADS classification, Ha EJ et al. concluded that ATA guidelines afford relatively moderate sensitivity for thyroid cancer detection, compared to the 2016 KTA (Korean Thyroid Association) and 2017 ACR guidelines. This limitation could arise due to the examined population—different region (different biology of the thyroid disease) and different hospitals (differences in equipment).

This work has some limitations. Firstly, we had a high percentage of malignant lesions, 32% higher than normal prevalence in population. That could have some impact on our statistics. That is why further prospective exploration of our results is needed. Secondly, we used strain elastography that is considered more operator dependent and requires more experience for accurate interpretation. Both this technique together with shear wave elastography are recommended by World Federation of Ultrasound in Medicine and Biology according to stratification of thyroid nodules (34). Thirdly, it should be noted that the possibility of false negatives was not completely avoidable, as patients with Bethesda Category II and no changes in B-mode US examination within 1 year did not undergo surgery.

## Conclusions

Our work has demonstrated that three B-mode features, such as composition (solid or >50% solid), shape (taller-than-wide) and halo pattern (without halo or partial halo), correspond to an intermediate risk of malignancy (TIRADS 3). The highest risk factor for cancer malignancy (TIRADS 5), turned out to be associated with margin (ill-defined) as the strongest single feature. A high risk of malignancy (TIRADS 4) was associated with echo pattern (markedly hypoechoic), microcalcifications (present), thyroid capsule (infiltration), macrocalcification (present), and Asteria scale (4 degree). There has been disagreement regarding the integration of sonoelastography into the TIRADS system in previous classifications. In our study, sonoelastography increases the predicted risk of malignancy especially in nodules in TIRADS 4 and 5 categories. Asteria 4 as a solitary feature in a solid tumor could result in its categorization as TIRADS 4 category, but coexistence with high risk features allows it to be upgraded to TIRADS 5 category. Lesion in TIRADS 5 category and suspicious sonoelastography (Asteria 3,4) indicates a highly increased risk of malignancy.

## Ethics Statement

This study was carried out in accordance with the recommendations of the Institutional Review Board of the Maria Sklodowska-Curie Institute - Cancer Center, Warsaw, Poland (MSCI), and all subjects gave written informed consent in accordance with the Declaration of Helsinki.

## Author Contributions

K-DS contributed conception and design of the study, wrote the first draft and sections of the manuscript. MD contributed conception and design of the study, organized the database. BM, KM, and RS organized the database. BM wrote sections of the manuscript. AK wrote sections of the manuscript. EB-Z organized the pathological analysis. AK organized the database. ZA contributed conception of the study. All authors contributed to manuscript revision, read, and approved the submitted version.

### Conflict of Interest Statement

The authors declare that the research was conducted in the absence of any commercial or financial relationships that could be construed as a potential conflict of interest.
